# Protective effects of celery (Apium Graveolens) on testis and cauda epididymal spermatozoa in rat

**Published:** 2014-05

**Authors:** Wesam Kooti, Esrafil Mansouri, Maryam Ghasemiboroon, Mahmoud Harizi, Ashraf Amirzargar

**Affiliations:** 1 *Department of Laboratory Sciences, School of Paramedicine, Student Research Committee, Ahvaz Jundishapur University of Medical Sciences, Ahvaz, Iran.*; 2 *Department of Anatomical Sciences, Cellular and Molecular Research Center, School of Medicine, Ahvaz Jundishapur University of Medical Sciences, Ahvaz, Iran.*; 3 *Department of Public Health, School of Health, Ahvaz Jundishapur University of Medical Sciences, Ahvaz, Iran.*; 4 *Department of Physiology, School of Medicine, Ahvaz Jundishapur University of Medical Sciences, Ahvaz, Iran.*

**Dear Editor**

Infertility is one of the health problems that will have a negative impact on the individual, social and economic and is seen in 10-15% of couples (1). About 40 % of the issues involved with infertility are due to the man. Male sperm cells count nowadays has decreased dramatically in comparison with those who lived 50 years ago (2). Causes of infertility in men are included: oligozoospermia, immaturity of sperm, sperm deformity, and sperm non-motility. Spermatogenesis takes place within the testes under control of testosterone secreted by the testes and secretory activity of the testes controlled by the hypothalamic-pituitary-testicle axis. Due to adverse effects and side effects of chemical drugs today, the use of traditional medicine, especially herbal therapy is taken into consideration. In traditional medicine, it has been pointed to therapeutic properties of celery. Celery has anti-fungal, anti-bacterial, and anti-cancer properties (3). Also this plant is an appetite stimulant and sexual booster (4). Previous studies have shown that sperm cells are largely vulnerable to oxidative stress but celery is rich in antioxidant compounds such as flavonoids (apiin and apigenin), vitamins E and C that can reduce oxidative stress (5, 6). So, in the present study the protective effect of celery was investigated on the cauda epididymal spermatozoa and testis in rat. 

A total number of 32 male Wistar rats (weighting 170-220 g) were prepared from animal house central of Ahvaz Jundishapur University of Medical Sciences. Animals were maintained in plastic cages with 12/12 h light/dark cycle at 21±2^o^C. All experimental animals were carried out in accordance with Ahvaz University Ethical Committee. Hydro-alcoholic extract of celery was prepared by maceration method. The rats were divided into four groups of 8 animals each: control, did not receive anything; vehicle, received propylene glycol; experimental groups, and received hydro-alcoholic extract of celery with doses of 100 and 200 (mg/kg) with solvent of propylene glycol by gavage once every 48 hours for twenty days. At the end of 20th day, rats were scarified under ketamine and xylazine anesthesia then the epididymis and testes were carefully separated. The epididymis was used for sperm count and testes were prepared for morphometric and histologic evaluation. Statistical significance of differences were assessed with one-way ANOVA by SPSS for windows (version 15) followed by LSD test. P<0.05 was assumed as statistically significant. Results of morphometric studies indicated a decrease in number of primary spermatocytes, Sertoli cells and sperm as well as an increase of lumen diameter of seminiferous tubules in vehicle group when compared to the control (p<0.05), but there was not different between the experimental groups and control (p>0.05). Evaluation of tissue sections showed that germinal epithelium in the control group was normal and tissue damage was not observed in epithelial tissue. However, in the vehicle group, epithelium was destroyed and arrangement of epithelial cells was disordered, and fluid aggregation is seen into the epithelial cell and also, reduction of epithelium thickness was observed. In the experimental group (100 mg/kg), there was arrangement in germinal epithelium cells but fluid aggregation was observed into the epithelial cells. A reduced epithelial thickness was seen only in some tubules. However, all these histological changes were less than the vehicle group. In the experimental group (200 mg/kg), tissue destruction was largely improved, and there was an arrangement of the epithelial cells, there was not fluid aggregation into the epithelium, and the thickness of the epithelium was returned almost too normal state. The results of the present study showed that hydro-alcoholic extract of celery improved the destructive effects of propylene glycol on the testes and sexual cells. These findings are similar to previous studied (7, 8). 

Previous studies have demonstrated that excessive alcohol consumption in men can cause a deficiency in testosterone production and testicular atrophy. Testicular atrophy results primarily from the loss of spermatogenic cells of the seminiferous tubules that this can be caused by oxidative stress generated by alcohol (9). Researches also indicated alcohol with involvement of phase system and activation of caspases induced apoptosis in testicular cells (10). Spermatogenesis and maturation of sexual cells depends on protection of cytotoxic and pathologic lesions that threatens these events. Free radicals due to a strong desire to get electrons induce damage to molecules such as fatty acid of biological membranes and its oxidation. Celery is a strong antioxidant due to flavonoids such as apiein and apigenin (5, 6). Antioxidant compounds are able to protect cell membranes against damage. Antioxidants directly or indirectly impact on hypothalamic-pituitary-testicular axis thus increase sperm count and fertility (5, 6). So celery can be considered as a medicinal herb for infertility. However, further clinical studies are recommended.

The study in the form of a research plan was approved with no 91s8 of Research Deputy of Ahvaz Jundishapur University of Medical Sciences. Finally, we acknowledge deputy vice-chancellor for research affairs of AJUMS for financial support, and particularly Research Consultation Center (RCC) for technical support.
